# Outcome and Survival Analysis of Multicenter Lung Metastasectomy for Primary Liver Tumor with Pulmonary Metastasis

**DOI:** 10.3390/cancers16173007

**Published:** 2024-08-29

**Authors:** Yu-Cheng Chang, Xu-Heng Chiang, Yu-Ting Tseng, Shuenn-Wen Kuo, Pei-Ming Huang, Mong-Wei Lin, Hsao-Hsun Hsu, Jin-Shing Chen

**Affiliations:** 1 Department of Surgery, National Taiwan University Hospital, National Taiwan University College of Medicine, Taipei 100225, Taiwan; richardchang@ntuh.gov.tw (Y.-C.C.); shjiang@ntuh.gov.tw (X.-H.C.); shuenn@ntuh.gov.tw (S.-W.K.); phuang3@ntuh.gov.tw (P.-M.H.); joehsu@ntuh.gov.tw (H.-H.H.); chenjs@ntu.edu.tw (J.-S.C.); 2 Department of Medical Education, National Taiwan University Hospital, Taipei 100225, Taiwan; 3 Department of Surgery, National Taiwan University Hospital Yun-Lin Branch, National Taiwan University College of Medicine, Yun-Lin 632, Taiwan; y00059@ms1.ylh.gov.tw; 4 Department of Surgery, National Taiwan University Hospital Hsin-Chu Branch, National Taiwan University College of Medicine, Hsin-Chu 302058, Taiwan; 5 Department of Surgical Oncology, National Taiwan University Cancer Center, Taipei 10672, Taiwan

**Keywords:** pulmonary metastasectomy, liver cancers, lung metastasis, survival, risk factors

## Abstract

**Simple Summary:**

We retrospectively evaluated the clinical outcomes of lung metastasectomy in 147 patients with pulmonary metastases from primary liver tumors at three medical centers. Multivariate analysis demonstrated that surgical resection as the initial primary liver tumor treatment and lower MELD-Na scores significantly correlated with better OS. These findings can guide thoracic surgeons in patient selection and predicting surgical outcomes.

**Abstract:**

Oligopulmonary metastases from primary liver tumors are typically treated surgically. We evaluated the clinical outcomes after lung metastasectomy in patients with pulmonary metastases from primary liver tumors. We retrospectively enrolled 147 consecutive patients with lung metastases from liver cancer who had undergone pulmonary metastasectomies at three medical centers between February 2007 and December 2020. All patients were pathologically confirmed to have lung metastases from liver cancer. Among the 147 patients, 110, 17, and 20 initially underwent surgical resection, radiofrequency ablation, and transcatheter arterial embolization, respectively. The 5-year overall survival (OS) in the study cohort was 22%. Univariate analysis revealed four factors associated with better OS: surgical resection as the initial primary liver tumor treatment (*p* = 0.004), a disease-free interval exceeding 12 months after the initial liver surgery (*p* = 0.036), a lower Model for End-Stage Liver Disease (MELD)-Na score (≤20) for liver cirrhosis (*p* = 0.044), and the absence of local liver tumor recurrence at the time of pulmonary metastasectomy (*p* = 0.004). Multivariate analysis demonstrated that surgical resection as the initial primary liver tumor treatment and lower MELD-Na scores significantly correlated with better OS. Our findings can assist thoracic surgeons in selecting suitable patients for surgery and predicting surgical outcomes.

## 1. Introduction

Primary liver malignancy, predominantly hepatocellular carcinoma, is characterized by its aggressive nature and high mortality rates when metastasis occurs [1,2]. Advances in primary local control in the liver through surgical and non-surgical approaches, such as radiofrequency ablation or transarterial embolization, have significantly contributed to the improved survival of patients with primary liver malignancies in the past [3]. However, the challenge lies in achieving effective control over extrahepatic metastases, as these currently prevent improvements in overall survival (OS).

Among extrahepatic metastases, lung metastasis is most frequent in patients with liver cancer. Enhancing the control of lung metastases could potentially be a pivotal factor in improving OS rates. A review of the recent literature indicated that the outcomes were not conclusive [4,5]. Several factors influencing these outcomes have emerged from the literature review, including liver tumor recurrence during lung metastasectomy, the number of extrahepatic metastatic sites, remission status in the liver prior to pulmonary metastasectomy, and the liver tumor-related disease-free interval [5].

The primary objective of this study was to assess perioperative and survival outcomes after pulmonary metastasectomy in patients with liver malignancies and pulmonary metastases.

## 2. Materials and Methods

### 2.1. Study Population

**Figure 1:**
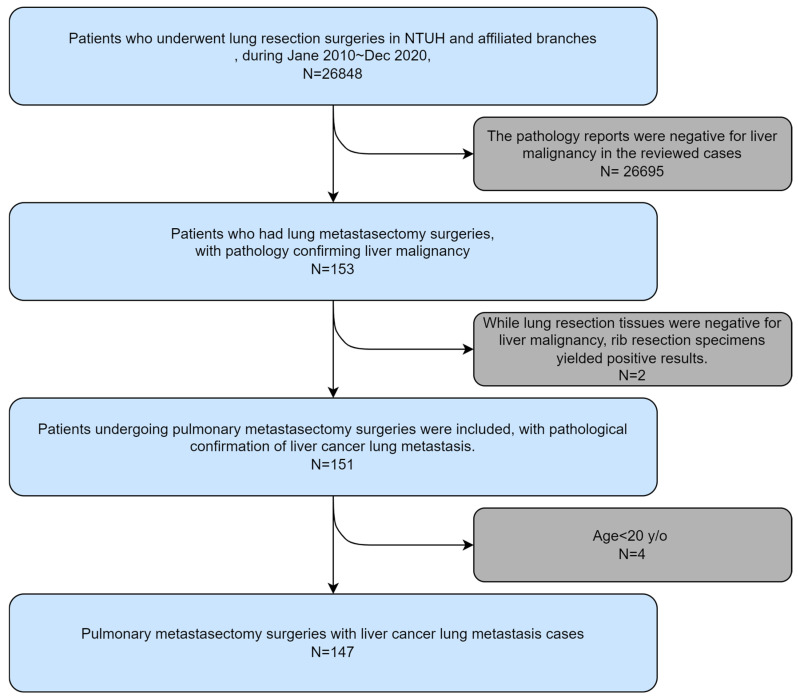
Algorithm for patient selection.

The subjects were recruited from the National Taiwan University Hospital, from January 2014 to December 2020 at the National Taiwan University Hospital Yun-Lin Branch, and from November 2014 to December 2020 at the National Taiwan University Hospital Hsin-Chu Branch (Figure 1).

The inclusion criteria were as follows: (1) patients aged > 20 years; (2) patients with a confirmed pathological diagnosis of primary liver malignancy; (3) patients who underwent primary management for liver tumors, including surgical resection or non-surgical interventions, such as radiofrequency ablation or transarterial embolization; and (4) patients who required lung resection as a metastasectomy for lung metastatic lesions. The exclusion criterion was a pathological report indicating no evidence of primary liver malignancy with lung metastasis.

All surgical procedures were performed at the National Taiwan University Hospital and its associated branches, including Yun-Lin and Hsin-Chu.

### 2.2. Study Patient Characteristic Factors

Regarding the patients’ baseline characteristics, we gathered information on factors such as the age at which patients underwent lung metastasectomy, sex, ECOG performance status during lung metastasectomy, and smoking history. Given that individuals with hepatitis may face an increased risk of developing liver cancer, we treated the hepatitis status as an independent variable. In terms of underlying health conditions, we documented the presence or absence of diabetes mellitus, hypertension, end-stage renal disease, and cardiac disease (as indicated in the outpatient clinic records). These factors were categorized as either “yes” or “no”.

Given that the initial cancer stage might correlate with prognosis, we included the initial Barcelona Clinic Liver Cancer classification (BCLC) stage before liver cancer treatment as a reflection of liver cancer status. We also assessed the preoperative status prior to lung metastasectomy, considering factors such as the alpha-fetoprotein level, aspartate aminotransferase, alanine aminotransferase, serum albumin level, international normalized ratio (INR), and the presence or absence of hepatic encephalopathy or ascites. Based on recent studies, both TAE/RFA and surgical resection are viable options for treating liver cancer, demonstrating similar efficacy in many cases. This is particularly relevant for patients who are not ideal candidates for surgery due to various health reasons [6]. Therefore, our study included both surgical resection and TACE/RFA in the management of primary liver malignancies. Regarding synchronous tumors, we meant to indicate that when lung metastasis occurs, it is accompanied by liver recurrence. In contrast, the other two patients had lung metastasis in conjunction with bone metastasis but showed no evidence of liver recurrence. Therefore, a distinction should be made between patients with stage IV disease and those with synchronous tumors.

Lung metastasectomy refers to the surgical resection of lung metastases of liver cancer. These metastatic sites could be solitary or multiple, and we classified the resection of all lung tumors detected through imaging as total lung tumor resection. In cases where any known lesion remained, it was categorized as incomplete resection. The details of the surgical approach were documented for subsequent subgroup analyses. These included parameters such as single-wedge or multiple-wedge resection, single-port VATS use, non-intubation surgery, operative time, operative blood loss, post-operative intensive care unit (ICU) stay, post-operative pleural drainage duration, instances of conversion from VATS to thoracotomy surgery, and whether prolonged air leakage occurred. Since lung cancer surgery is elective, most attending physicians at our institution recommend staging the procedures as two separate surgeries. This approach involves waiting for the patient to recover and be discharged after the first surgery before proceeding with the second. Typically, the second surgery is scheduled within approximately one month to balance treatment efficacy with the risks associated with performing both procedures simultaneously.

Pathological characteristics provide insights into the composition of metastatic lesions. We utilized tumor descriptors such as sample margins, uppermost tumor diameter, whether N1 or N2 mediastinal lymph node dissection was conducted, and whether these lymph nodes were involved. Regarding the resection margin, we define it as the pathological report of the tissue resected during VATS surgery.

All cases were managed with control of the primary liver malignancy. The lung metastasis interval was defined as the duration between the management of the primary liver lesions and the first date of detection of pulmonary metastasis. We also noted instances of liver cancer recurrence and documented metastatic sites beyond the lungs. Unilateral and bilateral lung metastases were also recorded.

Due to potential follow-up losses in outpatient clinics, we considered the last recorded Outpatient Department follow-up date as the most reliable survival endpoint. Most patients expired.

### 2.3. Statistical Analysis

Categorical variables are presented as numbers (percentages), while continuous variables are presented as means (standard deviations). Descriptive statistics are shown as mean ± standard deviation, and count (percent) for categorical variables. Both overall and disease-free survival were analyzed and calculated using the Kaplan–Meier method. Multivariate analysis was performed using the Cox regression model, and statistical significance was set at *p* < 0.05. Statistical analyses were performed using the IBM SPSS Statistics 26.0. software (IBM SPSS Inc., Chicago, IL, USA).

## 3. Results

### 3.1. Clinical Features

Between February 2007 and December 2020, 147 patients from a single institute and their affiliated branches were included in this study. Patients’ details are summarized in Table 1. All patients were diagnosed with a primary liver or lung malignancy with confirmed pulmonary metastasis. All patients underwent pulmonary metastasectomy via VATS or a thoracotomy approach. In our study, VATS accounted for approximately 90% (132 of 147 cases) of metastasectomy procedures. Patients who underwent VATS without lung resection were excluded. The distribution of the number of resected lung nodules was as follows: 51 patients had a single nodule removed, 32 had two nodules, 25 had three nodules, 13 had four nodules, and 26 had more than five nodules. This totals 147 nodules, which aligns with the cohort size.

The mean age of our study group was 58 years. Males were predominant. The vast majority of patients had an ECOG score of 0. About 63% of the study patients had been diagnosed with viral hepatitis. Just more than half of the patients had been diagnosed with hepatitis B virus, although some had hepatitis C (7.5%), while two patients were co-infected with hepatitis B and C. More than one-third of patients had underlying comorbidities. About three-fourths were non-smokers.

For primary liver malignancy management, about three-fourths received surgical intervention while the others received non-surgical intervention.

### 3.2. Pathological Features

Pathological features of the patients are summarized in Table 2. The pathological reports revealed hepatocellular carcinoma and intrahepatic cholangiocarcinoma in our patients. Most cases were resected with a negative margin in pulmonary metastasectomies. Lymph node dissection was performed in less than one-third of patients, of whom only eight were shown to have pathological lymph node involvement. In addition, there were two cases of cholangiocarcinoma in our series. Patient 1 was a 57-year-old male, a hepatitis B carrier, who underwent liver surgery as the primary treatment for liver cancer. The initial pathological stage was pT1N0M0. This patient also had diabetes mellitus (DM) and hypertension, which were managed with medication. He underwent a pneumonectomy, with an overall survival of 16 months and a disease-free survival of 6 months. Patient 2 was a 57-year-old female who also received liver surgery for liver cancer, with an initial pathological stage of pT2aN0M0. She underwent multiple lung wedge resections, resulting in an overall survival of 31 months and a disease-free survival of 2 months. Both cases involved surgery, including hepatectomy and lung metastasectomy.

### 3.3. Perioperative Outcome

Perioperative data on the patients are summarized in Table 3. In most of our pulmonary metastasectomy patients, low blood loss, short chest tube indwelling time, and short postoperative hospital stay, as well as short ICU stay, were noted. Among the 147 patients, two deaths occurred on postoperative day 30. In one patient, extensive liver tumor invasion had occurred through the diaphragm, with direct invasion into the lung parenchyma. The patient underwent thoracoabdominal incision for tumor excision, which was accompanied by a large amount of blood loss intraoperatively. The patient collapsed in the ICU after surgery. Electrocardiography showed pulseless electrical activity. The patient received cardiopulmonary resuscitation, with the return of spontaneous circulation after 20 min. The other patient had an underlying thromboembolism and underwent recurrent liver tumor and lung metastasectomy during the same surgery; then, this patient died after experiencing ischemic stroke on post-operative day 2.

### 3.4. Factors Correlated with Overall Survival

The 5-year OS rate was 22% in these 147 patients. According to univariate analysis, the OS, as analyzed by Kaplan–Meier curves, was significantly better for patients positive for the following factors: surgical resection as the initial treatment of primary liver tumor, disease-free interval after liver surgery over 12 months, liver tumor recurrence at the time of pulmonary metastasectomy, and MELD_Na score < 20 (Table 4).

**Figure 2:**
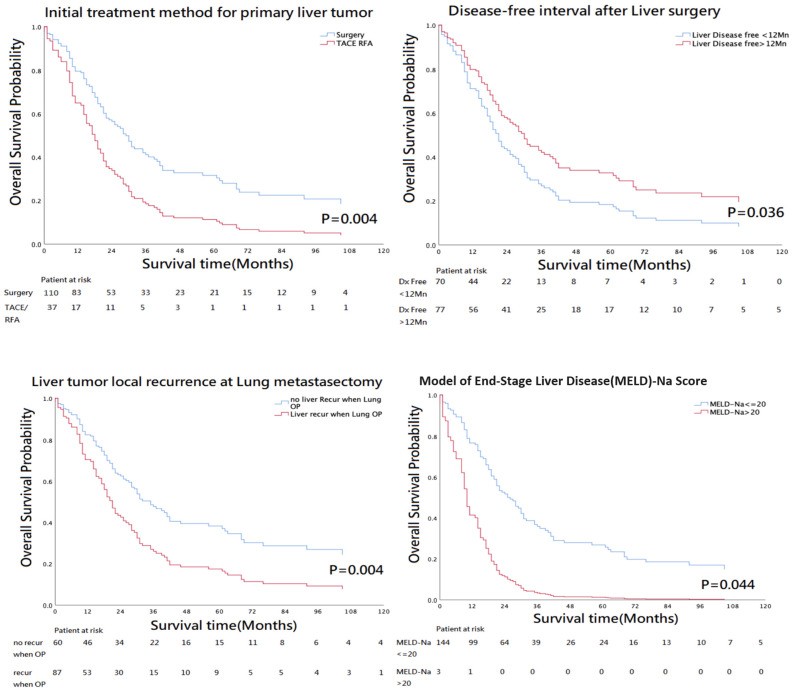
Univariate analysis revealed four factors significantly correlated with overall survival: surgical resection as the initial primary liver tumor treatment; disease-free interval exceeding 12 months after the initial liver surgery; absence of local liver tumor recurrence; MELD-Na score lower than 20. However, multivariate analysis showed only surgical resection as initial treatment of the primary liver tumor and MELD_Na score below 20 as factors significantly correlated with overall survival.

Multivariate analysis revealed that the only significant factors correlated with OS were surgical resection as the initial treatment of the primary liver tumor and a MELD_Na score < 20 (Figure 2; Table 5).

## 4. Discussion

Pulmonary metastasectomy has been suggested as the first-line treatment for patients with liver malignancies with lung metastasis [1], if it is resectable. We sought to determine the factors associated with OS in these patients. In the univariate analysis, we found that surgical resection as the initial primary liver tumor treatment, a disease-free interval exceeding 12 months after the initial liver surgery, a MELD-Na score ≤ 20 for liver cirrhosis, and the absence of local liver tumor recurrence at the time of pulmonary metastasectomy were significant predictors of survival. Multivariate analysis demonstrated that surgical resection as the initial primary liver tumor treatment and MELD-Na scores correlated significantly with OS.

In previous studies [5,7,8,9,10], some factors predicting OS have been reported, including liver tumor recurrence at the time of lung metastasectomy, number of metastatic sites, remission status of the liver cancer [11], distant metastasis-free interval, liver tumor disease-free interval, and history of liver tumor recurrence during pulmonary resection. Taken together with our univariate analysis results, surgical resection as the initial treatment of the primary liver tumor and liver tumor recurrence status at the time of pulmonary metastasectomy may have a stronger influence on OS.

All stages of liver malignancy can progress to pulmonary metastasis. Among the 147 patients in our study, the percentage of the initial TNM stages of liver cancer was stage 1, 19.0%; stage 2, 22.4%; stage 3, 25.2%; and stage 4, 7.5%. Additionally, because of this retrospective data harvesting, there is inevitably some data loss many years later, as about 25.9% of TNM data could not be clarified. However, we found no significant correlation between the initial TNM stage and OS in patients who underwent pulmonary metastasectomy. This may be due to the occurrence of lung metastasis, and the stage of liver cancer shifted to stage 4. No significant differences were found between groups.

To evaluate the prognosis of a malignancy, we anticipated that a shorter period of time before the occurrence of metastasis might lead to a worse prognosis. However, when we divided the patients into two groups based on whether the period of liver-to-lung metastasis was more or less than 1 year, we found no statistically significant difference (*p* = 0.130). Therefore, a shorter period of liver-to-lung metastasis could not predict worse OS in our patients.

We also applied the BCLC stage as a preoperative evaluation method. Most of our patients had better liver function, namely, stage A. This may be because most pulmonary metastasectomies are selective. Surgery was preferred in patients whose liver function was within the normal range. The BCLC stage was not significantly associated with the OS.

Generally, from an oncological point of view, a complete resection of all tumors, if resectable, is believed to be related to better oncological outcomes. We applied this concept to lung metastases in patients with liver cancer. If the primary tumor of the liver malignancy was under control, a lung metastasis should be considered and treated as a primary tumor. There may be a single metastasis, oligometastases, or even multiple metastatic tumors. We compared OS according to whether complete resection of all detected lung nodules was performed but found no significant difference (*p* = 0.544). We then performed an analysis considering the actual number of resected nodules. We divided patients into two groups according to whether resection of more than three lung nodules was performed, but again found no evidence of a significant difference (*p* = 0.256). Thus, lung metastasis from liver cancer cannot simply be considered as a primary tumor. Complete resection of the lung nodules and the number of resected lung nodules did not correlate with OS in patients who underwent lung metastasectomy.

Only one arm study was included. We were unable to compare patients with liver cancer with lung metastasis who were not treated with surgical intervention. Therefore, we had no data supporting a head-to-head comparison of lung metastasectomy and the influence thereof on OS in these patients. However, according to ASCO data, the 5-year survival rate is approximately 3% in liver cancer patients with lung metastasis who are not treated. Our data showed that liver cancer patients with lung metastasis who underwent lung metastasectomy showed a 5-year survival rate of more than 22%. However, variations among study patients may play a role [12,13]. The OS of patients who underwent lung metastasectomy tended to be better than that of patients who did not receive any treatment. Nevertheless, further studies are needed to explore this trend further.

Taken together, complete resection of lung metastasis showed no better OS benefit, and metastasectomy patients seemed to have a better 5-year OS. The explanation for this phenomenon may be that, when performing metastasectomy, we tend to perform lung resection of the largest lesion. The scenarios were as follows: if one lung metastasis was present, metastasectomy had only one goal; if two lung metastases were present, the larger one would be resected in all cases, irrespective of whether the smaller one was resected; and if multiple lung metastases were present, i.e., three or more nodules, an attempt was made to resect as much tumor volume as possible [14]. As surgical intervention for metastases may be effective in dealing with the largest lesions, a trend toward better OS was noted in patients who underwent lung metastasectomy. Accordingly, tumor burden reduction may play an important role in cancer lung metastasis outcomes. Further studies are necessary to allow better differentiation.

When dealing with primary tumors, surgeons perform lymph node dissection from an oncological perspective. In our study, whether lymph node dissection was performed showed no significance in terms of OS. However, during our data analysis, we found that the lymph node positivity rate was approximately 20%. Initially, we expected this rate to correlate with overall survival. Since the majority of cases did not involve lymph node dissection, our final analysis did not show a significant correlation with overall survival. (Please note that this analysis included only the 41 cases with lymph node data.) The initial analysis suggests that lymph node positivity could correlate with poorer overall survival outcomes. Assuming a 20% positivity rate, it is likely that around 30 out of the 147 patients might have had positive lymph nodes. We can only hypothesize that with a larger sample size of lymph node dissections, we might observe statistically significant differences. Currently, our data are insufficient to draw firm conclusions. Further studies are needed to confirm whether lymph node positivity is a significant prognostic factor. Future retrospective or prospective studies could provide valuable insights. If positive results are obtained, they could support the inclusion of lymph node dissection as part of the standard procedure for lung metastasectomy.

On the other hand, we also found that a disease-free interval of more than 12 months after liver surgery and an MELD_Na score < 20 were also correlated with better OS. The MELD_Na score was first prospectively developed and validated as a chronic liver disease severity scoring system that uses a patient’s laboratory values for serum bilirubin, serum creatinine, and the INR for prothrombin time to predict 3-month survival. Firstly, we attempted to determine whether cirrhosis was present, while pulmonary metastasectomy was one of the factors in OS. This was also significant in the univariate analysis based on cirrhosis. However, we depended only on imaging findings, such as CT and abdominal sonography, for the diagnosis of cirrhosis. More reliable data and more specific methods are needed to describe liver conditions before lung surgery. The MELD score was then considered, and the MELD-Na score, which was developed in 2016, was considered as a better predictor of liver disease conditions. Therefore, we used the MELD-Na score to analyze liver function in patients with metastatic liver malignancy.

This study had some limitations. Firstly, inherent biases associated with retrospective studies could not be avoided. We attempted to reduce these biases by using multivariate analysis. Secondly, the number of patients in the study was relatively small and non-randomized. Therefore, the data must be interpreted cautiously. Thirdly, only one patient arm was included in the study. We were unable to directly compare our patients with those who did not undergo surgical interventions. Additionally, our study spanned a longer period, which could influence survival outcomes due to advancements in diagnostic accuracy, surgical techniques and safety, pathological diagnostic precision, and the overall experience of the healthcare team. Finally, there were some missing data for several variables in this study, which may confound the results and limit the conclusions that can be drawn. These missing data introduce potential bias and should be considered when interpreting our findings.

Although the extended duration of our study introduces several limitations, future research could benefit from larger datasets and longer follow-up periods to better validate the influence of additional factors. This study had several strengths, including that this research stands as one of the largest cohort studies investigating the impact of lung metastasectomy on the survival rates of patients battling metastatic liver cancer.

## 5. Conclusions

In conclusion, two significant factors were associated with an improved OS after pulmonary metastasectomy in liver cancer patients with pulmonary metastases. Surgical hepatectomy as the primary treatment for initial liver tumors emerged as a significant predictor of enhanced survival outcomes. Additionally, a MELD-Na score < 20 at the time of pulmonary metastasectomy was identified as another factor associated with better OS. Importantly, as pulmonary metastasectomy for primary liver tumors with lung metastasis has been shown to be both effective and safe, with patients experiencing short hospital stays and minimal time spent in the ICU, this may be a suitable treatment for these patients.

## Figures and Tables

**Table 1:** Demographic and clinical features of primary liver malignancy patients with lung metastasis patients who underwent metastasectomy.

	n (%) or Mean ± SD (Range)
Total Patients	147 (100%)
Age, yr	58.4 ± 11.5 (33–84)
Male	121 (82.3%)
ECOG	
0	142 (96.6%)
≥1	5 (3.4%)
Smoking status	
Smoker	31 (21.1%)
Non-smoker	116 (78.9%)
Viral hepatitis	
Absent	53 (36.1)
Present	94 (63.9)
HBV	81 (55.1)
HCV	11 (7.5)
HBV + HCV	2 (1.3)
Comorbidities	57 (38.8)
DM	31 (21.1)
HTN	38 (25.9)
ESRD	7 (4.8)
Cardiac diseases	9 (6.1)
Initial TNM stage of primary liver tumor ^a^	
I	28 (19.0)
II	33 (22.4)
III	37 (25.2)
IV	11 (7.5)
Loss data	38 (25.9)
Initial BCLC stage of primary liver tumor ^a^	
A	36 (24.5)
B	52 (35.4)
C	11 (7.5)
Loss data	48 (32.6)
Initial treatment method for primary liver tumor	
Surgery	110 (74.8)
Non-surgery	37 (25.2)
RFA ^b^	17 (11.6)
TAE ^b^	20 (13.6)

^a^ In total, 18 and 9 patients lacked the TNM stage and BCLC stage, respectively; ^b^ 2 patients received RFA and TACE. Abbreviations: BCLC, Barcelona Clinic Liver Cancer staging; DM, diabetes mellitus; ECOG, Eastern Cooperative Oncology Group performance status; ESRD, end-stage renal disease; HBV: hepatitis B virus; HCV: hepatitis C virus; HTN: hypertension; RFA: radiofrequency ablation; SD, standard deviation; TAE: transarterial embolization, including TACE: transarterial chemoembolization; yr, years old.

**Table 2:** Clinical features at the time of lung metastasectomy and pathological features of primary liver tumor patients with lung metastasis patients who underwent metastasectomy.

	n (%) or Mean ± SD (Range)
Total patients	147 (100.0%)
Disease-free interval after liver surgery (mo)	22.3 ± 28.7 (0~206)
Synchronous tumor (liver, lung)	9 (6.1)
Non-synchronous tumor	138 (93.9)
Liver-to-lung interval (mo)	36.4 ± 40.4 (1~232)
Liver tumor local recurrence at the time of lung metastasectomy	84 (57.1)
Variables at the time of lung metastasectomy	
Child–Pugh class	
Child A	142 (96.6)
Child B	5 (3.4)
Abnormal AFP ^a^	75 (51.0)
Abnormal Bilirubin ^b^	37 (25.2)
Abnormal AST/ALT ^b^	66 (44.9)
Abnormal INR level ^b^	10 (6.8)
MELD-Na score	8.79 ± 3.3 (6.4~26.4)
Complete resection of all metastatic nodules	84 (57.1)
Number of resected metastatic nodules	
1	51 (34.7)
2	32 (21.8)
3	25 (17.0)
4	13 (8.8)
≥5	26 (17.7)
Maximum size of the metastatic nodules (mm)	20.0 ± 13.9 (0.4 mm~85 mm)
Laterality	
Unilateral metastasis	68 (46.3)
Bilateral metastasis	79 (53.7)
Resection margin ^a^	
Positive	7 (4.8)
Negative	126 (85.7)
Loss data	14 (9.5)
Lymph node metastasis	
No LND	106 (72.1)
Perform LND	41 (27.9)
Positive	8 (5.5)
Negative	33 (22.4)

^a^ In total, 5 and 10 patients lacked the preoperative serum AFP level and resection margin, respectively. ^b^ Mean and standard deviation for Bilirubin 0.81 ± 0.34; AST 38.3 ± 21.4; ALT 39.1 ± 28.6; INR level 1.23 ± 1.45. Abbreviations: AFP, alpha-fetoprotein; AST, aspartate aminotransferase; ALT, alanine aminotransferase; INR, international normalized ratio; LND: lymph node dissection; SD, standard deviation.

**Table 3:** Perioperative outcomes of primary liver tumor patients with lung metastasis patients who underwent metastasectomy.

	n (%) or Mean ± SD (Range)
Total patients	147 (100%)
Surgical method	
Sublobar resection	127 (86.4)
Lobectomy	18 (12.2)
Bilobectomy, pneumonectomy	2 (1.4)
Approach method	
Thoracotomy	15 (10.2)
VATS	132 (89.8)
Uniportal VATS	44 (29.9)
Sequential metastasectomy for bilateral metastasis	5 (3.4)
Non-intubated anesthesia	11 (7.5)
Dye localization ^a^	18 (12.2)
Operative time, min	119.3 ± 75.1 (30~407)
Operative bleeding, mL	22.1 ± 93.2 (minimum~800)
Conversion	0
Length of hospital stay, day	5.0 ± 4.7 (1~41)
Postoperative ICU stay, day	0.7 ± 1.5 (0~14)
Chest tube duration, day	2.6 ± 2.7 (0~18)
Morbidities	13 (8.8)
Minor	10 (6.8)
Prolonged air leak	5 (3.4)
Arrhythmia	2 (1.4)
Subcutaneous emphysema	1 (0.7)
Hemothorax	0
Chylothorax	0
Hoarseness	0
Significant pleural effusion ^b^	1 (0.7)
Urinary retention	2 (1.4)
Major	2 (1.4)
Stroke	1 (0.7)
PEA, CPR	1 (0.7)
30-day mortality	2 (1.4)

^a^ Computed tomography-guided tattooed tumor localization; ^b^ significant pleural effusion: daily drainage more than 200 mL. Abbreviations: ICU, intensive care unit; SD, standard deviation; VATS, video-assisted thoracoscopic surgery.

**Table 4:** Univariate analysis of correlations between clinicopathological features and OS for primary liver tumor patients with lung metastasis patients who underwent metastasectomy.

Variables	Patient Number	Hazard Ratio	95% CI	*p* Value
Age, yr				
<65	99	1		
≥65	48	0.78	0.520–1.172	0.231
Sex				
Female	26	1		
Male	121	1.101	0.653–1.859	0.718
ECOG				
0	142	1		
≥1	5	1.154	0.365–3.648	0.808
Smoking status				
Non-smoker	116	1		
Smoker	31	1.256	0.735–2.146	0.405
Viral hepatitis				
Absent	53	1		
Present	94	0.913	0.613–1.360	0.655
Comorbidities				
Absent	90	1		
Present	57	0.609	0.462–1.029	0.069
Initial TNM stage of primary liver tumor ^a^				
I–II	51	1		
III–IV	48	1.298	0.809–2.081	0.279
Initial BCLC stage of primary liver tumor ^a^				
A–B	88	1		
C	11	0.904	0.258–3.172	0.335
Initial treatment method for primary liver tumor				
Surgery	110	1		
Non-surgery	37	1.897	1.229–2.929	0.004
Disease-free interval after liver surgery				
<12 months	70	1.521		
≥12 months	77	1	1.027–2.251	0.036
Liver-to-lung interval				
<12 months	48	1		
≥12 months	99	1.373	0.911–2.07	0.130
Liver tumor local recurrence at the time of lung metastasectomy				
Absent	62	1		
Present	85	1.818	1.207–2.736	0.004
MELD-Na score				
≤20	144	1		
>20	3	3.372	0.093–0.096	0.044
AFP level ^a^				
Normal	55	1		
Abnormal	75	1.202	0.799–1.807	0.378
Complete resection of all metastatic lung nodules				
No	63	1.13		
Yes	84	1	0.761–1.679	0.544
Number of resected metastatic nodules				
1–2	83	1		
≥3	64	0.793	0.532–1.183	0.256
Maximum size of the metastatic nodules				
<2 cm	83	1		
≥2 cm	62	0.964	0.649–1.433	0.857
Unilateral or bilateral lung metastasis				
Unilateral	68	1		
Bilateral	79	1.312	0.888–1.940	0.173
Surgical method				
Sublobar resection	127	1		
Lobectomy,	18	1.265	0.175–9.125	0.816
≥Bilobectomy	2	0.617	0.77–4.921	0.649
Approach method				
Thoracotomy	15	1		
VATS	132	1.400	0.765–2.563	0.276
LN dissection				
No	106	1		
Yes	41	1.363	0.499–3.725	0.545
Margin ^a^				
Free	126	1		
Involvement	7	0.906	0.332–2.473	0.847

^a^ In total, 18, 9, 5, and 10 patients lacked the TNM stage, BCLC stage, preoperative serum AFP level, and Margin, respectively. Abbreviations: AFP, alpha-fetoprotein; BCLC, Barcelona Clinic Liver Cancer staging; CI, confidence interval; ECOG, Eastern Cooperative Oncology Group performance status; LN, lymph nodes; OS, overall survival; VATS, video-assisted thoracoscopic surgery.

**Table 5:** Multivariate analysis of correlations between clinicopathological features and OS for primary liver tumor patients with lung metastasis patients who underwent metastasectomy.

Variables	Hazard Ratio	95% CI	*p* Value
Initial treatment method for primary liver tumor			
Surgery	1		
Non-surgery	1.865	1.177–2.958	0.008
Disease-free interval after liver surgery			
<12 months	1		
≥12 months	0.674	0.446–1.019	0.061
Liver tumor local recurrence at the time of lung metastasectomy			
Absent	1		
Present	1.393	0.894–2.171	0.143
MELD-Na score			
≤20	1		
>20	3.977	1.188–13.316	0.025

Abbreviations: CI, confidence interval; OS, overall survival.

## Data Availability

All data generated or analyzed during this study are included in this published article.
